# Differential Regulation of Genes Involved in Root Morphogenesis and Cell Wall Modification is Associated with Salinity Tolerance in Chickpea

**DOI:** 10.1038/s41598-018-23116-9

**Published:** 2018-03-19

**Authors:** Mayank Kaashyap, Rebecca Ford, Himabindu Kudapa, Mukesh Jain, Dave Edwards, Rajeev Varshney, Nitin Mantri

**Affiliations:** 1 School of Science, The Pangenomics Group, RMIT University, Melbourne, Australia; 2 School of Natural Sciences, Environmental Futures Research Institute, Griffith University, Queensland, Australia; 3 International Crops Research Institute for the Semi-Arid Tropics (ICRISAT), Hyderabad, India; 4 National Institute of Plant Genome Research, New Delhi, India; 5 School of Plant Biology, The University of Western Australia, Perth, Australia

## Abstract

Salinity is a major constraint for intrinsically salt sensitive grain legume chickpea. Chickpea exhibits large genetic variation amongst cultivars, which show better yields in saline conditions but still need to be improved further for sustainable crop production. Based on previous multi-location physiological screening, JG 11 (salt tolerant) and ICCV 2 (salt sensitive) were subjected to salt stress to evaluate their physiological and transcriptional responses. A total of ~480 million RNA-Seq reads were sequenced from root tissues which resulted in identification of 3,053 differentially expressed genes (DEGs) in response to salt stress. Reproductive stage shows high number of DEGs suggesting major transcriptional reorganization in response to salt to enable tolerance. Importantly, cationic peroxidase, Aspartic ase, *NRT1/PTR*, phosphatidylinositol phosphate kinase, *DREB1E* and *ERF* genes were significantly up-regulated in tolerant genotype. In addition, we identified a suite of important genes involved in cell wall modification and root morphogenesis such as dirigent proteins, expansin and casparian strip membrane proteins that could potentially confer salt tolerance. Further, phytohormonal cross-talk between *ERF* and *PIN-FORMED* genes which modulate the root growth was observed. The gene set enrichment analysis and functional annotation of these genes suggests they may be utilised as potential candidates for improving chickpea salt tolerance.

## Introduction

Chickpea is the second most important grain legume crop grown in the tropics and sub-tropics for its high nutritional value and natural ability to fix atmospheric nitrogen^1^. Over the past 30 years, chickpea production has increased from 6.4 to 13.1 million metric tons (FAOSTAT, 2013). Major producers are India, Australia, Turkey and Myanmar who collectively contribute more than 75% of the global production. From 2009 to 2013, the global area under chickpea increased from 11.5 to 13.5 million hectares and production increased by 25% (from 10.4 to 13.1 million metric tons) (FAOSTAT, 2013). Given its high nutritional content, market value, adaptability and nitrogen fixation ability, chickpea is being increasingly recognised as a global staple food crop of the future.

Salinity is a major problem for crop production especially in the arid and semi-arid regions where chickpea is mainly cultivated^2,3^. Chickpea is challenged by a number of abiotic stresses but salinity is a major constraint that alone accounts for 8–10% of crop yield losses worldwide^4,5^. Chickpea has a narrow genetic base, however, considerable genetic variability has been observed amongst the germplasm in response to salt stress^5–8^.

In order to harness this genetic variation for crop improvement, it is crucial to unravel the molecular mechanisms and potential candidate genes that would allow overcoming its phenotypic plasticity.

Salt stress causes accumulation of Na^+^ and Cl^−^ ions in various plant tissues and impairs the plant growth especially during the reproductive stage^9^ Khan *et al*.^5,10^. Salinity disturbs the cellular ionic and osmotic environment within the plant. This severely affects important processes like photosynthesis and other important metabolic processes leading to retarded plant growth and poor reproductive success^11,12^. Based on a number of physiological studies in chickpea, researchers have proposed accumulation of ions in various tissues as detrimental to crop productivity^13,14^ Pushpavalli *et al*.^1^). However, there is little knowledge on the effect of salinity on various developmental stages in chickpea. Further, salt tolerance mechanisms such as ion-exclusion, vacuolar compartmentalization and detoxification that are reported in other crops have not been well studied in chickpea^15–17^.

Several studies have reported that salt stress induces complex regulatory mechanisms through major transcriptional reorganisation^18–21^. To understand this, many candidate genes have been identified in response to salt stress through evolving high-throughput approaches like microarrays, SuperSAGE, and deep sequencing^22,23,21^. Considerable efforts were made to map the tolerance trait and identify the genes underlying the tightly linked DNA markers in JG 11 × ICCV 2 population^1^. However, full gene information could not be retrieved due to lack of chickpea reference genome at the time. In recent studies, salt responsive genes including cell wall biogenesis, heat shock proteins and transcription factors were identified to be differentially regulated between contrasting genotypes at various developmental stages^21,18^. Despite considerable effort, the *cis-acting* genes and their gene networks that actually regulate this multigenic trait could not be intuitively elucidated. In addition, important genes involved in morphogenesis and organ modulation resulting in salt stress avoidance or tolerance could not be underpinned in chickpea. Meanwhile, there has been considerable progress on improving salt tolerance in crops like wheat, rice, and soybean where identification and expression of potential candidate genes such as transmembrane ion-transporters led to improved tolerance. However such knowledge is lacking in chickpea. Therefore, the aim of this study was to identify the comprehensive set of genes involved in modulation of salt stress tolerance in chickpea at various developmental stages.

## Results and Discussion

### Phenotypic Response to Salt Stress

#### Phenotypic Characteristics at the Vegetative Stage

**Figure 1:**
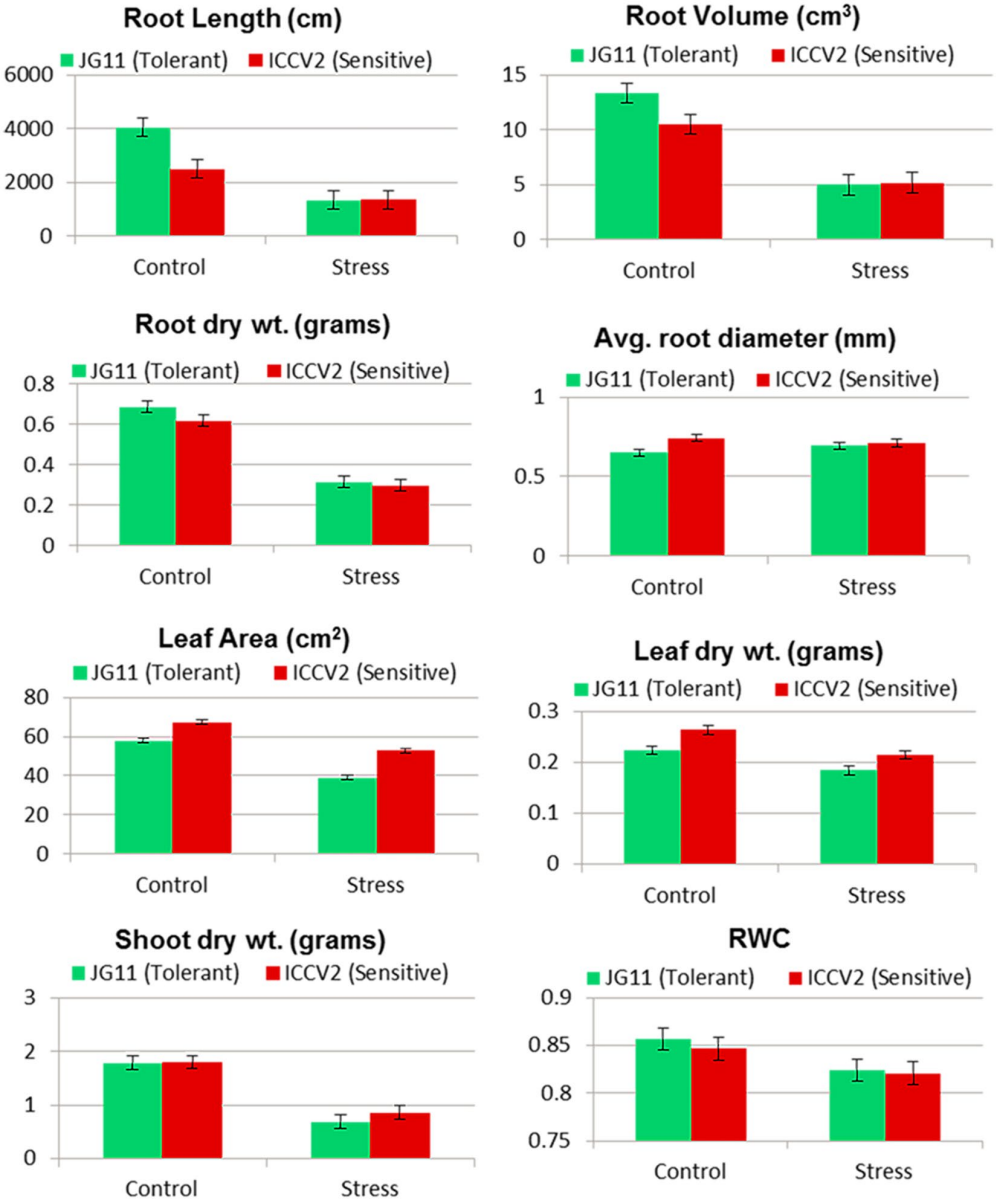
Physiological screening of chickpea genotypes in response to salt stress. Important parameters like root length, root biomass, root diameter, leaf area and shoot biomass were measured at the vegetative stage.

The root length of tolerant genotype was 50% higher as compared to the sensitive genotype in the control condition, however it reduced drastically by 70% in both the genotypes under salt stress (Fig. 1). Similarly, the root volume was 24% higher in the tolerant genotype in the control condition while it reduced by 70% in both genotypes under salt stress. Importantly, the tolerant genotype showed an increase in average root diameter as compared to the sensitive genotype. These results are in agreement to previous studies that reported agravitropic growth of roots in response to salt stress, i.e., growth in diameter rather than length^24^. The root dry weight, shoot dry weight and surface leaf area were almost same in both the genotypes in control condition while it reduced by 50% in both the genotypes in the stress condition.

#### Phenotypic Characteristics at the Reproductive Stage

**Figure 2:**
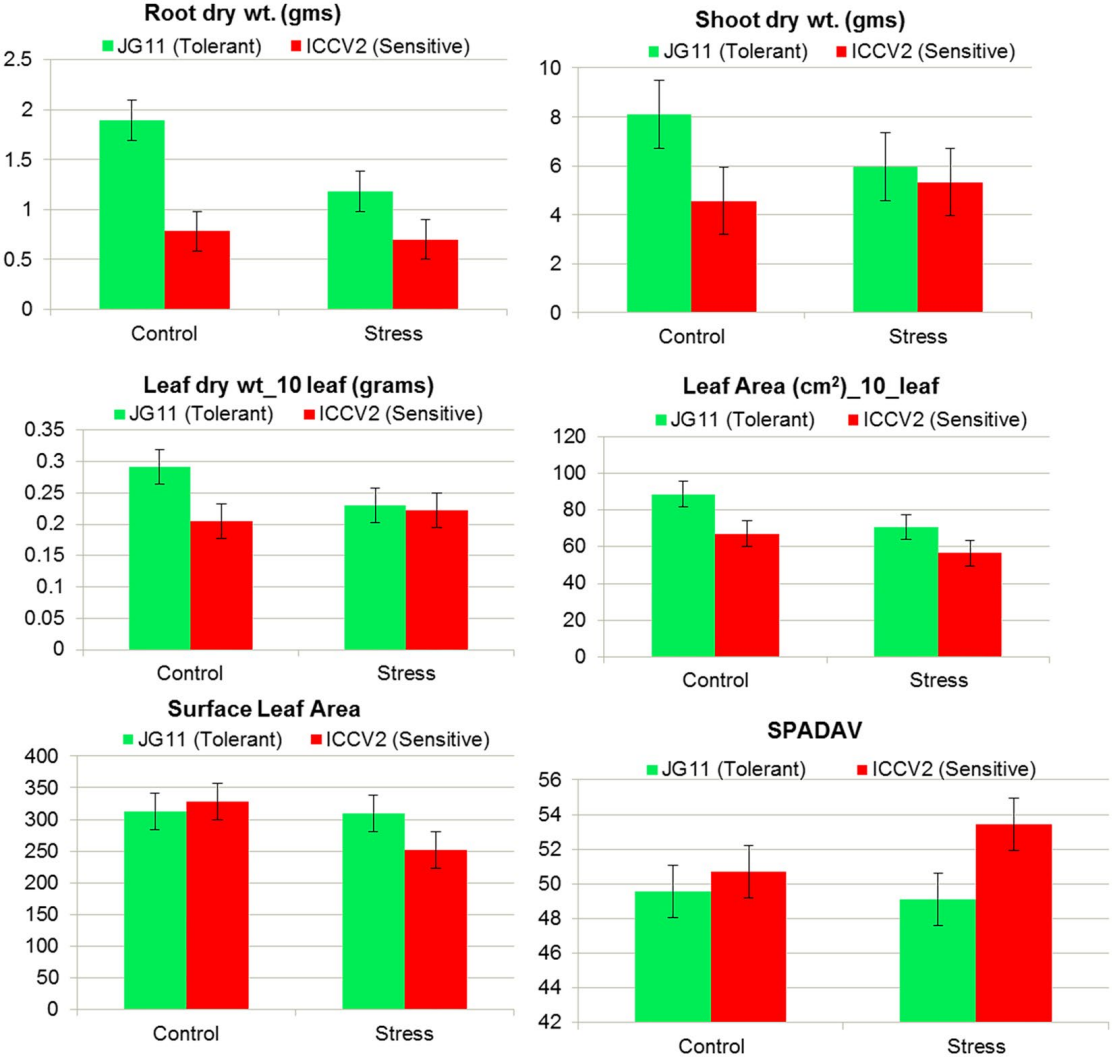
Physiological screening of chickpea genotypes in response to salt stress. Important parameters like root length, root biomass, root diameter, leaf area and shoot biomass were measured at the reproductive stage.

At reproductive stage, the root dry weight decreased by almost 50% in the tolerant genotype during stress while it was constant in the sensitive genotype (Fig. 2). This may suggest that ion accumulation in the sensitive genotype added to the weight in stressed condition while the tolerant genotype show decrease in root biomass through ion-exclusion. Interestingly, the shoot biomass also decreased by 25% in the tolerant genotype while it increased by 20% in the sensitive genotype which again could be due to higher absorption and accumulation of salt ions during the stress. In addition, leaf dry weight also decreased in the tolerant genotype while it increased in the sensitive genotype during the salt stress condition.

### RNA-sequencing, mapping and Gene Set Enrichment Analysis (GSEA)

#### Reference genome guided assembly of RNA reads

The root transcriptome profiles of JG 11 (salt tolerant genotype) and ICCV 2 (salt sensitive genotype) at the vegetative and reproductive stages were studied by RNA-sequencing. More than 480 million reads were generated from the three biological replicates of the two genotypes (2 genotypes × 2 time-points × 2 conditions × 3 biological replicates) with a library size of ~20 million single-end reads per sample. Approximately 92% reads passed the quality trimming and 72% clean reads mapped to the improved CDC frontier kabuli v2.6.3 reference genome (http://doi.org/10.7946/P2G596) using tophat2 (tophat-gcc/2.0.13). The accepted hits from this mapped assembly were used to identify the number of counts per gene in response to salt stress at the vegetative and reproductive stages of the tolerant and the sensitive genotypes. The reference-guided assembly produced 47,031 transcripts representing 35,855 gene loci which are 7% more than the reference annotation (Edwards *et al*., 2016). In addition to this, 3,912 potentially novel fragments which have at least one splice junction shared with a reference transcript were found. The fragments mapped against the reference to Exons (99.3%), Introns (0.66%) and Exon-Exon (0%) regions by their types. A gene was considered to be expressed if normalised counts of reads mapping to that gene(s) was greater than one. Using this cut-off, 21,622 gene loci were identified as expressed in at least one condition in response to salt stress. Out of the 33,351 total genes annotated in reference genome, 21,518 (64%) in ICCV 2 C, 21,250 (63%) in JG 11 C, 20,865 (62%) in ICCV 2 LC and 21,622 (64%) in JG 11 LC were considered as expressed for further differential gene expression analysis. Based on the different number of counts in a particular stress condition and developmental stage, these gene(s) were assigned as differentially expressed. To check if the gene expression difference is due to salt treatment, we established a hierarchical clustering amongst the three biological replicates of the control and stress conditions at the reproductive stage. The r-log transformation values of gene counts clustered the three biological replicates of each condition in one group. This also shows that the diversity amongst the samples is due to different gene expressions in response to salt stress and not due to slight differences in the experimental setup.

#### Identification of Differentially Expressed Genes (DEGs)

**Figure 3:**
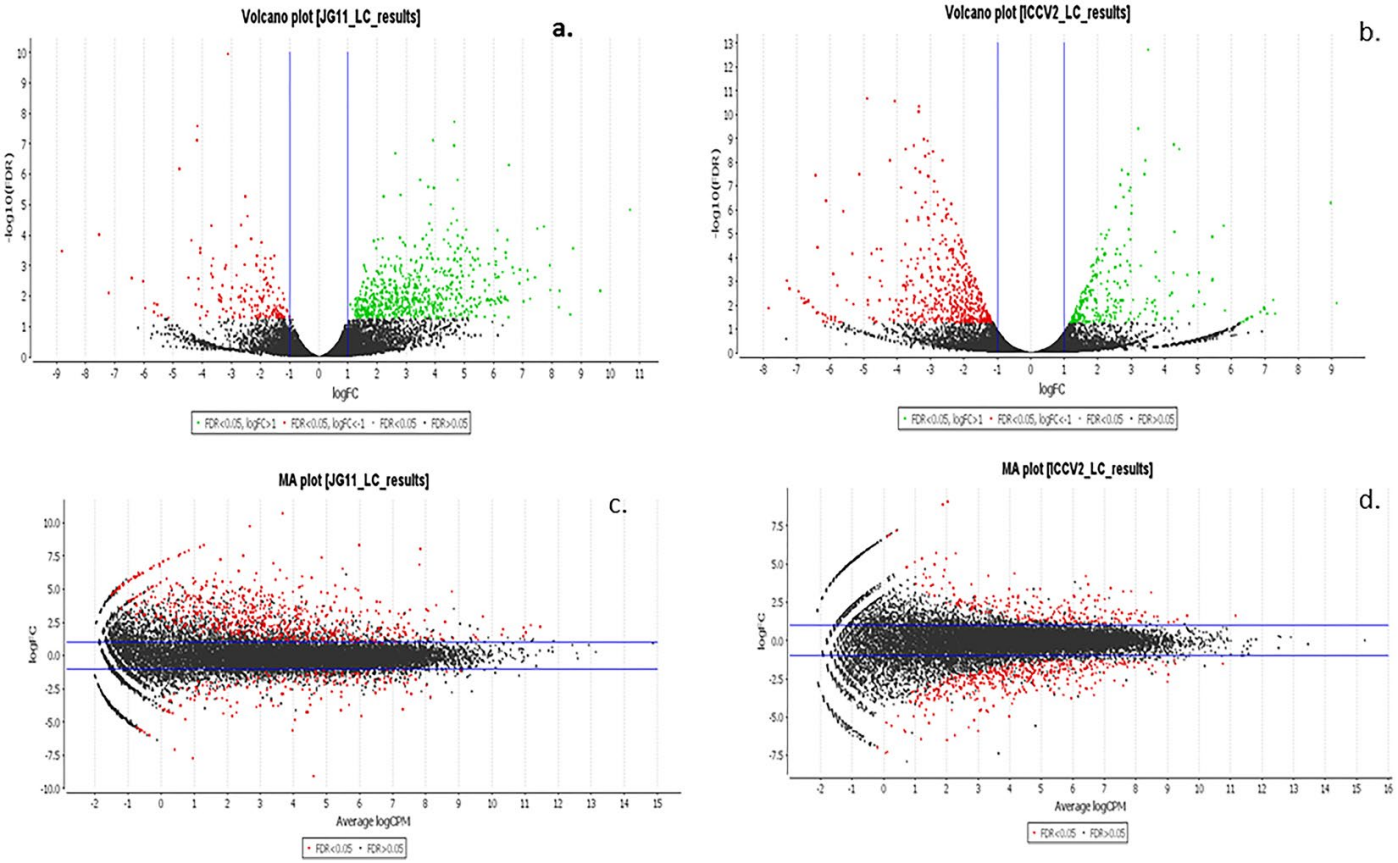
Volcano plot showing the significantly expressed differential genes in response to salt stress.

**Figure 4:**
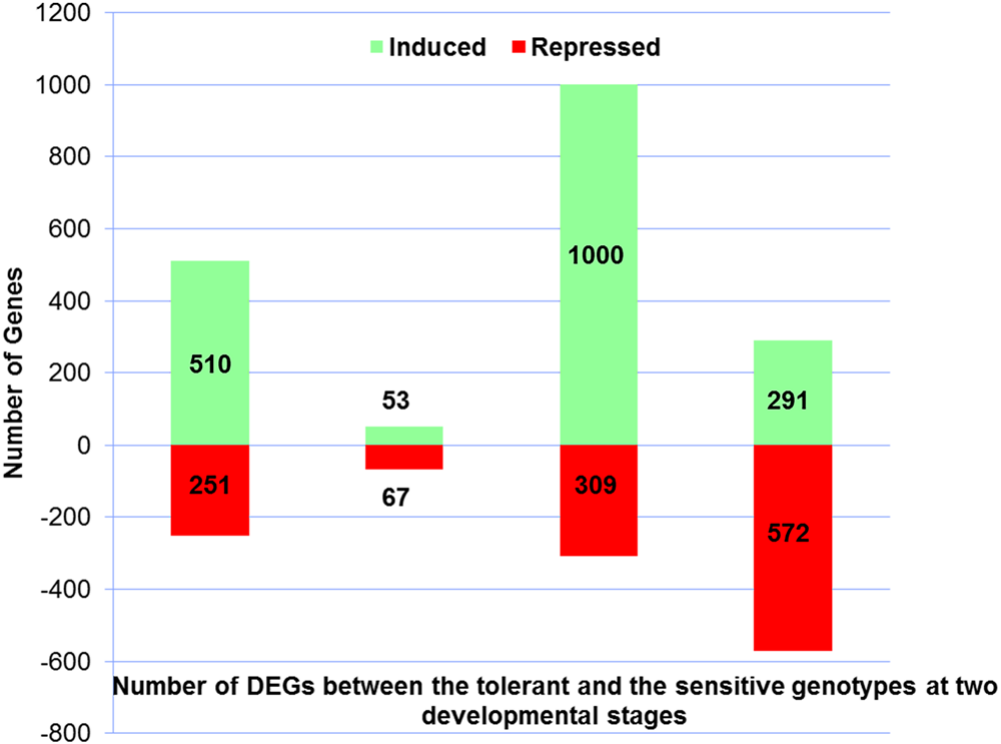
Significant Differentially Expressed Genes (DEGs) between the two genotypes at two developmental stages in response to the salt stress. More number of genes was up-regulated in the tolerant while more number of genes was down-regulated in the sensitive genotype.

**Figure 5:**
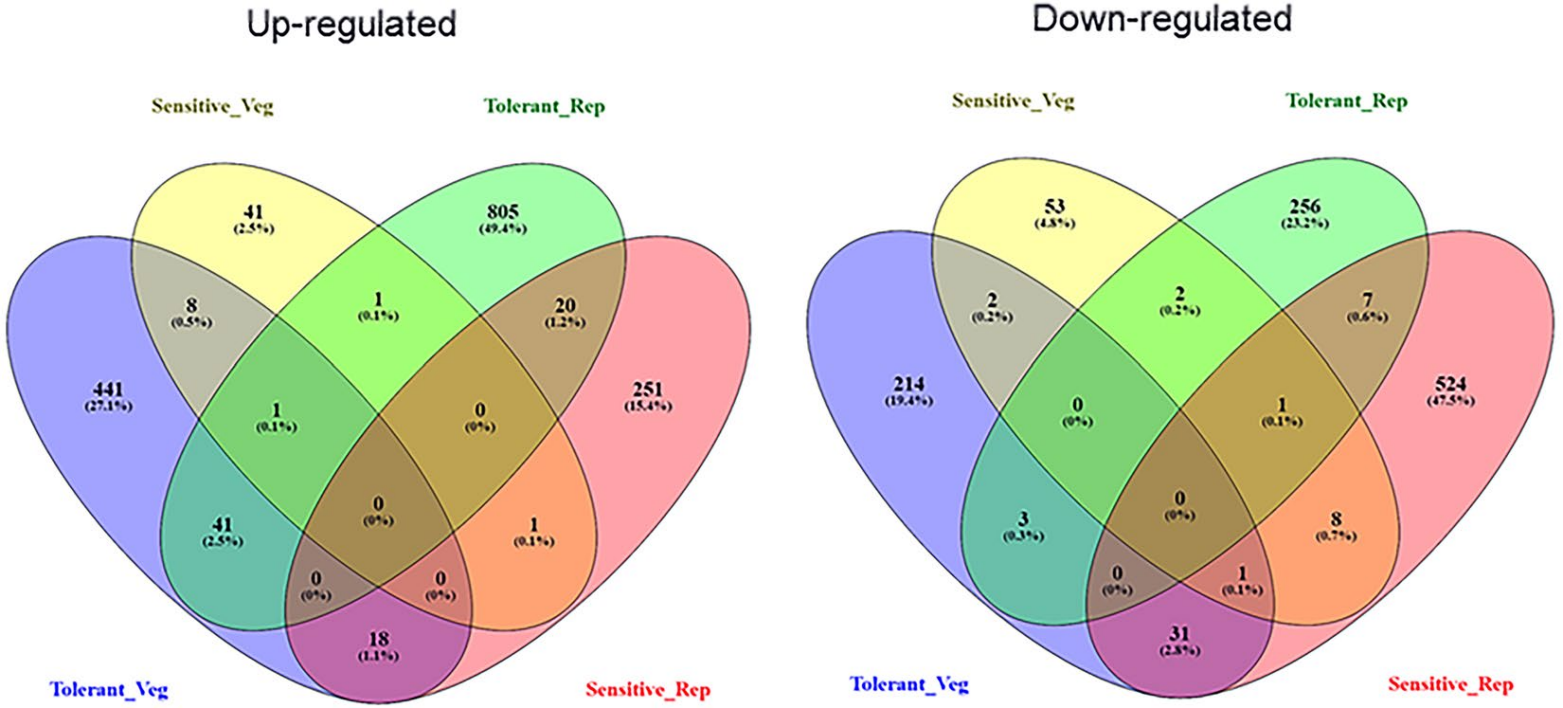
Venn diagram showing the commonly up- and down-regulated differentially expressed genes amongst the genotypes at the two developmental stages in response to the salt stress.

The gene-counts for the RNA-Seq libraries from the three biological replicates of the tolerant and the sensitive genotypes at the vegetative and reproductive stages were extracted using HTSeq counts. The log_2_ fold-change of gene-counts was calculated for the stress against control for the salt-tolerant (JG 11) and salt-sensitive (ICCV 2) genotypes and compared at the two developmental stages. EdgeR (GLM-likelihood ratio test) was performed using Blast2GO PRO software to identify the potential differentially expressed genes (fold change > 1 and FDR < 0.05) (Fig. 3). A total of 3,053 differentially expressed genes (DEGs) were identified amongst the two contrasting genotypes at the two developmental stages. Interestingly, the number of DEGs varied from 1,309 in the tolerant genotype at reproductive stage to 863 in the sensitive genotype at reproductive stage. At vegetative stage, the number of DEGs varied from 761 in the tolerant genotype to 120 in the sensitive genotype (Fig. 4). At the vegetative stage, the salt-tolerant genotype had more up-regulated genes (510) and less down-regulated genes (251) whereas the salt-sensitive genotype had less up-regulated genes (53) and more down-regulated genes (67). During the reproductive stage, the salt-tolerant genotype had more up-regulated genes (1000) and less down-regulated genes (309) whereas the salt-sensitive genotype had less up-regulated genes (291) and more down-regulated genes (572). In both the cases, the salt tolerant genotype possessed more number of total DEGs as compared to the salt-sensitive genotype. Both the genotypes show a large number of differentially expressed genes at the reproductive stage, suggesting a major transcriptional re-programming in response to salt stress at this important developmental stage. On comparing the commonalities amongst DEGs expressed in the salt-sensitive and salt-tolerant genotypes, 588 genes in JG 11-Veg, 74 genes in ICCV 2-Veg, 872 in genes JG 11-Rep and 612 genes in ICCV 2-Rep were exclusively expressed in the individual genotype/developmental condition (Fig. 5). In comparison, 144 DEGs were commonly expressed amongst ‘ICCV 2-Rep *vs*. JG 11-Rep’, 11 common DEGs in ‘ICCV 2-Veg *vs*. ICCV 2-Rep’, 17 common DEGs in ‘ICCV 2-Veg *vs*. JG 11-Rep’, and 57 common DEGs in ‘ICCV 2-Rep *vs*. JG 11-Veg’.

#### Mapping the functions of genes to GO categories and KEGG database

**Figure 6:**
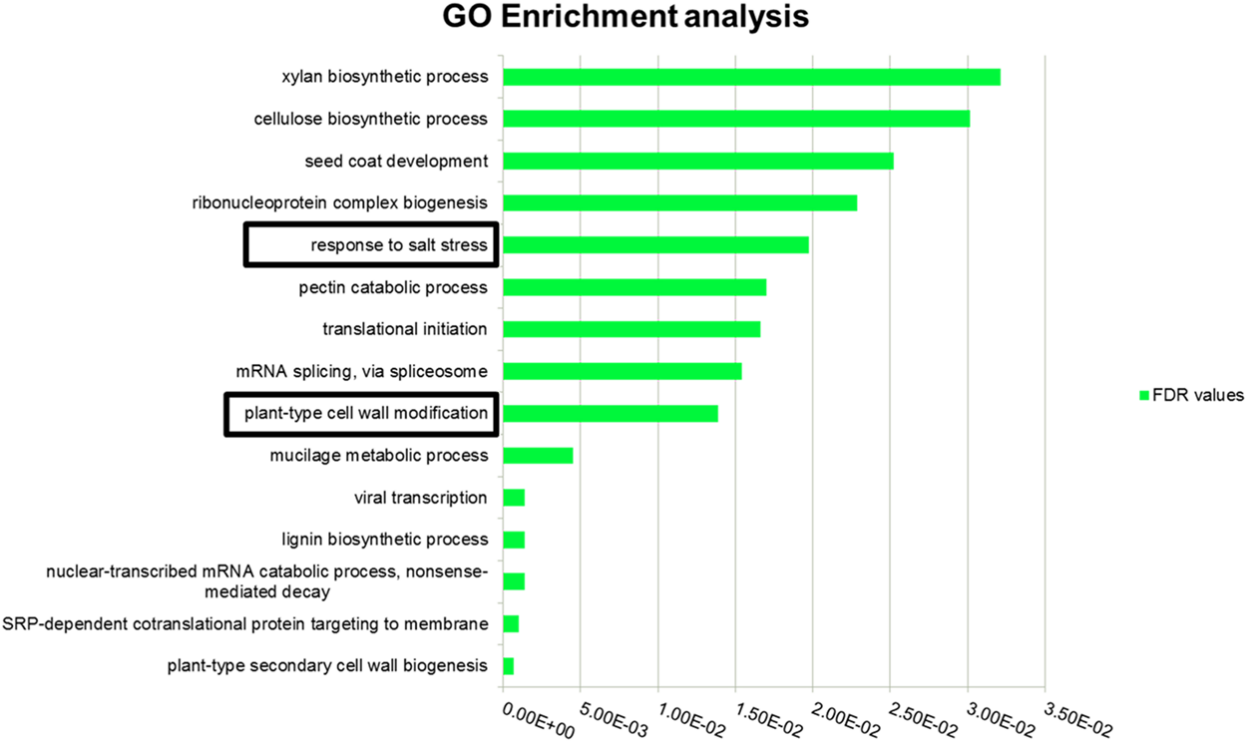
Gene set enrichment analysis shows enriched GO categories based on differentially expressed genes between tolerant and sensitive genotypes in response to salt stress.

The FASTA sequences of DEGs were BLAST searched against nr-databases to functionally annotate and map sequences to gene ontology GO terms using Bast2GO. From the total DEGs identified, only 76% (1,937) could be significantly annotated and mapped to molecular, cellular and biological GO categories. Subsequently, Gene Set Enrichment Test (GSEA) was performed for the induced/repressed genes using Fisher’s test (FDR < 0.05) to find the over-represented and under-represented categories (Fig. 6). Amongst these, biological processes like response to salt (GO: 0009651), plant-type secondary cell wall biogenesis (GO: 0009834), SRP-dependent co-translational protein targeting to membrane (GO: 0006614), mRNA splicing via spliceosome (GO: 0000398) and cellulose biosynthetic process (GO: 0030244) were significantly represented (Table 1). The KEGG pathway map analysis showed differentially expressed genes representing mainly glycerophosholipid metabolism, phenylpropanoid biosynthesis, inositol metabolism and starch metabolism.

**Table 1:** List of significantly enriched Gene Ontology based on Fisher’s Test (FDR < 0.05) amongst tolerant vs. sensitive genotype in response to salt stress.

GO-ID	Term	Over/Under
GO:0009651	Response to salt stress	OVER
GO:0006614	SRP-dependent cotranslational protein targeting to membrane	OVER
GO:0000184	Nuclear-transcribed mRNA catabolic process, nonsense-mediated decay	OVER
GO:0009809	Lignin biosynthetic process	OVER
GO:0019083	Viral transcription	OVER
GO:0010191	Mucilage metabolic process	OVER
GO:0009827	Plant-type cell wall modification	UNDER
GO:0000398	mRNA splicing, via spliceosome	OVER
GO:0006413	Translational initiation	OVER
GO:0045490	Pectin catabolic process	UNDER
GO:0022613	Ribonucleoprotein complex biogenesis	OVER
GO:0010214	Seed coat development	UNDER
GO:0030244	Cellulose biosynthetic process	UNDER
GO:0045492	Xylan biosynthetic process	UNDER
GO:0016072	rRNA metabolic process	OVER

#### Validation of RNA-Seq results with quantitative Real-Time PCR

**Figure 7:**
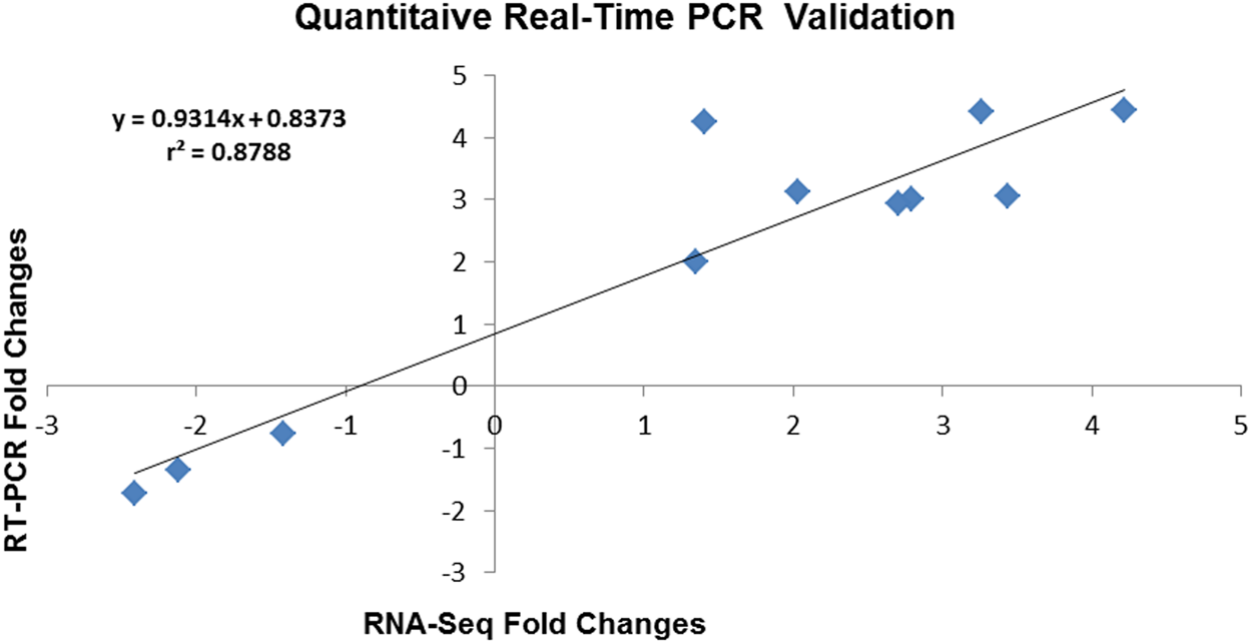
Quantitative Real-Time validation with 10 salt responsive genes with r2 > 0.87 as the significant threshold.

The reliability of RNA-Seq gene expression was conformed with 10 salt responsive genes including cation exchanger, blue copper, glutaredoxin, ascorbate oxidase and chloride-channel protein. Fold changes obtained from RT-PCR results correlated well with fold changes of cation exchanger, blue copper and glutaredoxin, calcium-transporting ATPase and ascorbate oxidase which confirms the validity of RNA-Seq data (Fig. 7).

### Transcriptomic responses to salt stress

#### Biological significance of DEGs in response to salt

**Figure 8:**
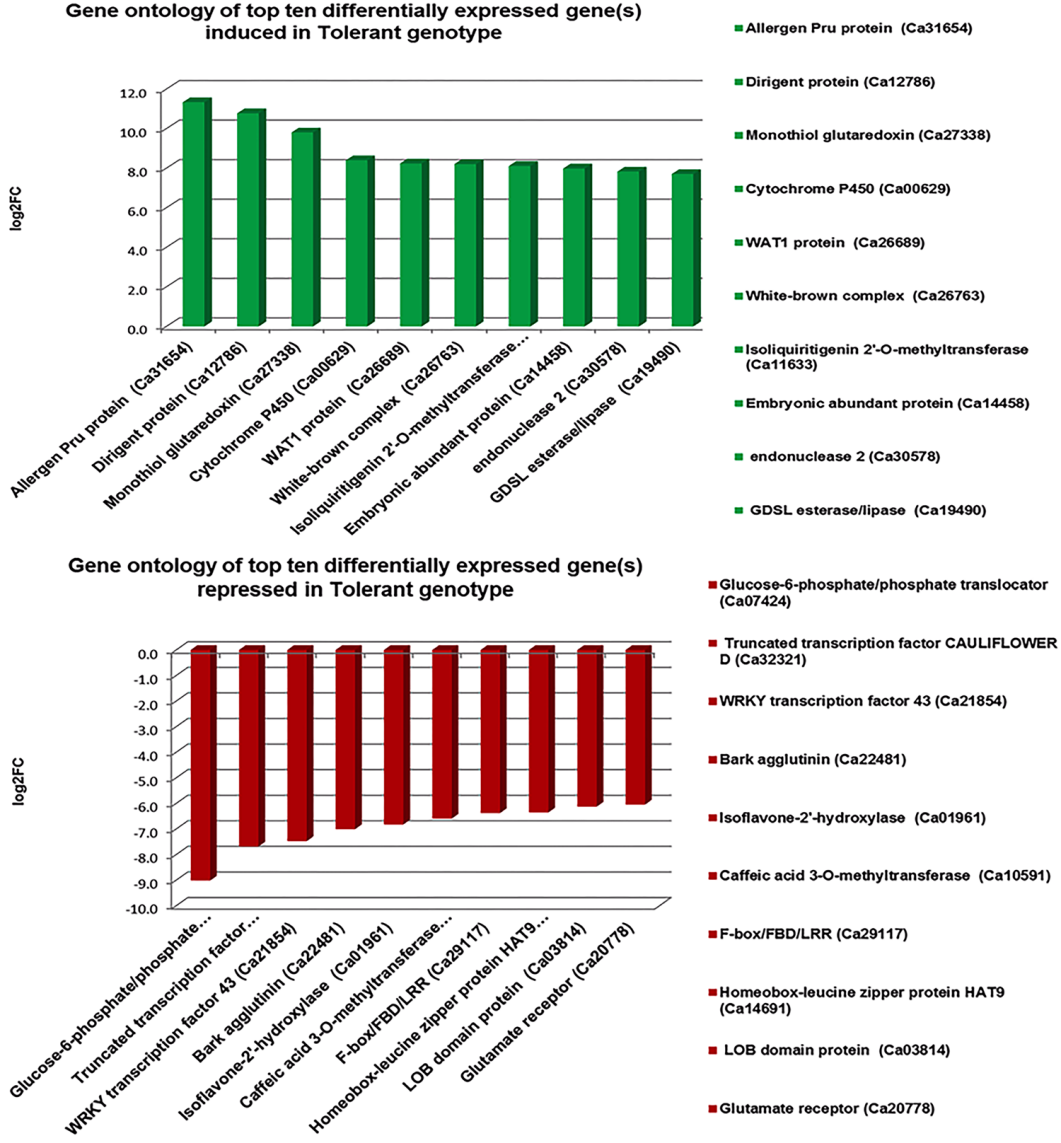
Top ten differentially expressed genes in the tolerant genotype in response to salt stress. The most important genes like dirigent proteins are up-regulated in the tolerant genotype while glucose-6-phospahte genes were highly down-regulated in response to the salt stress.

**Figure 9:**
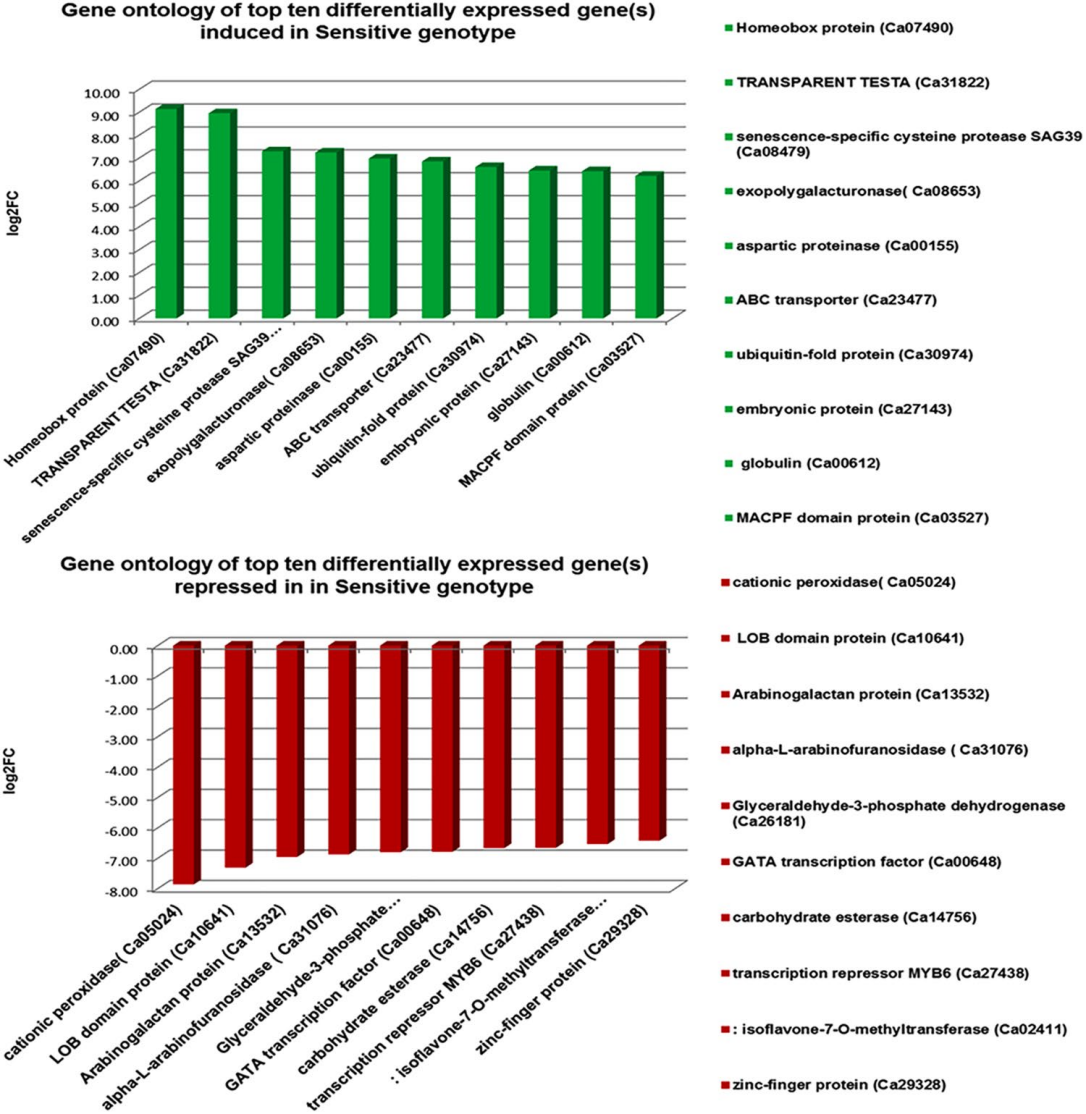
Top ten differentially expressed genes in the sensitive genotype in response to the salt stress. The most important genes like cationic peroxidase were highly down-regulated in response to the salt stress.

The genes that were significantly differentially expressed between the two genotypes at vegetative and reproductive stages were mainly associated with cell wall organisation, transmembrane transport, signal transduction, oxidative stress, transcriptional regulation, phytohormone signalling and root development (Fig. 8). In particular, majority of abiotic stress-related genes were significantly up-regulated in the tolerant genotype while down-regulated in the sensitive genotype under salt stress. These include lipid transfer proteins, *NAC* factors, sugar porters, *cation/H*^+^ exchangers, peroxidases, casparian strip membrane protein, root meristem development gene, cytochrome P450, Purple acid phosphatase, *AP2-ERF* factors, *LEA* proteins and auxin efflux carrier proteins. On the contrary, other genes such as cysteine-rich repeat secretory protein, pectin esterase, bidirectional sugar transporter, senescence-specific cysteine protease, abscisic acid hydroxylase and glutamate receptors were down-regulated in the tolerant genotype but up-regulated in the sensitive genotype (Fig. 9).

#### DEGs Involved In Sensory Mechanism for Cell Detoxification

**Figure 10:**
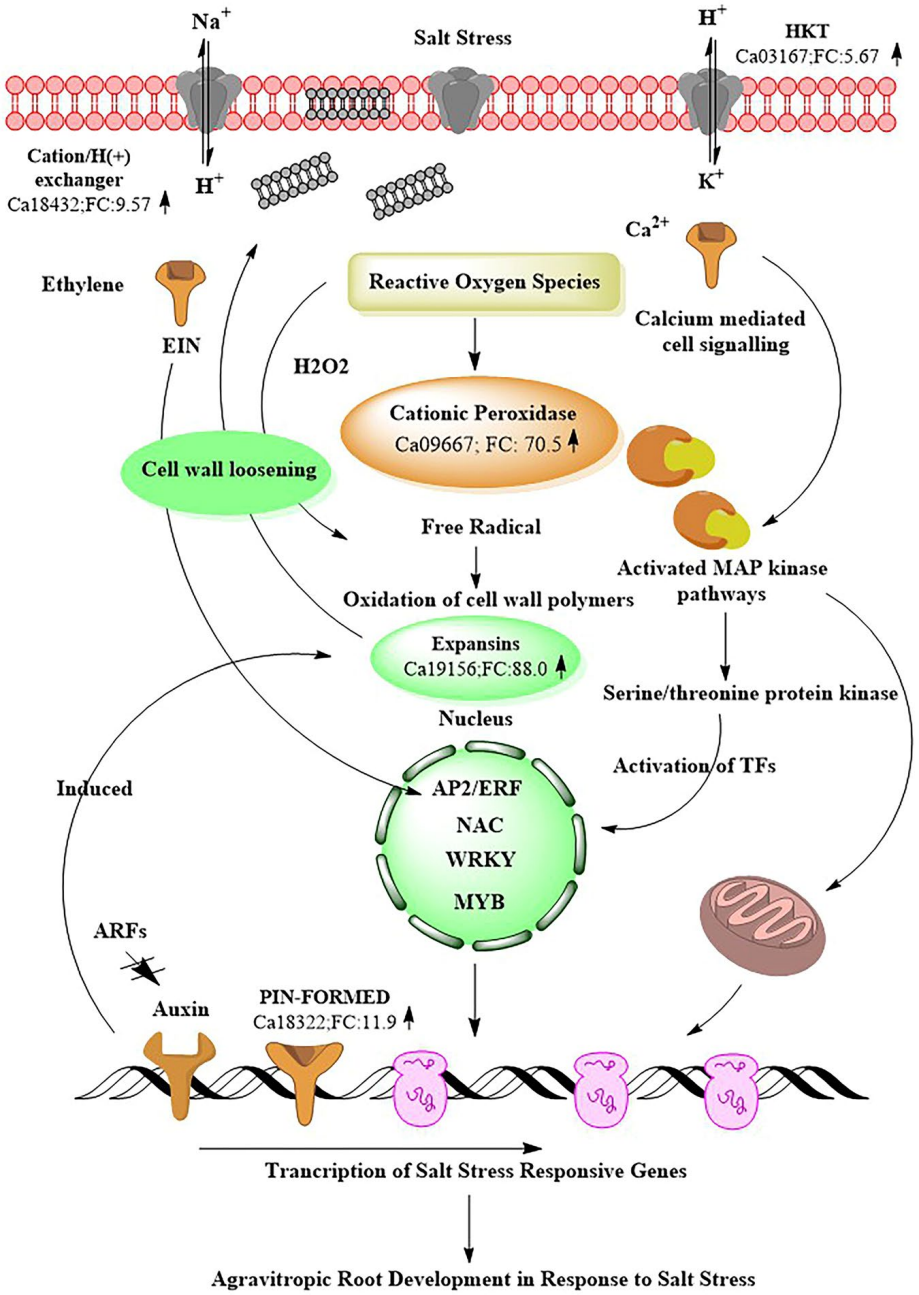
A schematic cell signalling cascade based on differential gene expression pattern in roots in response to the salt stress.

Plants are sessile organisms and have developed various sensory mechanisms to respond to abiotic stresses^25^. The first sensory mechanism when stress is perceived by the cell is an outburst of reactive oxygen species (*ROS*). These *ROS* instigate the H_2_O_2_ mediated detoxification and calcium-mediated activation of stress signalling pathways^26^. Further, the *ROS* activate the plasma membrane permeable calcium ion channels such as calcium-transporting *ATPase* which catalyse the Ca^2+^ induced cell signalling during salt stress^27,28^. Interestingly, calcium-transporting *ATPase* gene was highly induced in the tolerant genotype (Ca04200; FC: 18.50 ↑) while repressed in the sensitive genotype (Ca04200; FC: −3.89 ↓), which suggests that an efflux of Ca^2+^ is important for signalling sensory pathways (Fig. 10). Another calcium binding gene was significantly induced in the tolerant genotype (Ca07521, FC: 9.84 ↑) while repressed in the sensitive genotype (Ca07521, FC: −3.29 ↓).

The calcium ions in turn activate the protein kinase genes such as mitogen activated protein kinases (*MAPK*) and calcium dependent protein kinases (*CDPK*) which help in signal relay mechanism (Wang *et al*.^25^). The MAP kinase gene (Ca01482; FC: 5.46 ↑) expression, followed by *serine/threonine protein kinases* (Ca09102; FC: 16.5 ↑) were induced in the tolerant genotype while repressed in the sensitive genotype (Ca09102; FC: −2.69 ↓). Importantly, these protein kinases cause the phosphorylation/dephosphorylation of signal cascade proteins and activate various transcription factors which eventually regulate salt stress responsive gene expression^29^.

Upon successful signalling, hydrogen peroxide has to be disintegrated. This is mainly achieved through the activation of peroxidases. Peroxidases play a diverse role in plant physiology and defence response and apart from detoxification of H_2_O_2_, they are involved in lignin biosynthesis^30^. Several antioxidant genes such as cationic peroxidases were significantly induced in the tolerant genotype (Ca09667; FC: 70.5 ↑) while being highly repressed in the sensitive genotype (Ca05024; FC: −232.32 ↓) in response to salt stress (Table 2). Importantly, lignin-forming anionic peroxidase that detoxifies H_2_O_2_ and has a putative role in combating environmental oxidative stress was significantly up-regulated in the tolerant genotype (Ca02966: FC: 14.12 ↑).

**Table 2:** List of important differentially expressed genes in tolerant and sensitive genotypes in response to salt stress.

Gene ID	Gene Name	Tolerant genotype (Fold change)	Sensitive genotype (Fold change)
Ca19118	Acid beta-fructo furanosidase	150.59	−21.97
Ca31032	Pectate lyase *Medicago truncatula*	56.60	−12.91
Ca05594	2-oxoglutarate dependent dioxygenase *AOP1*	43.47	−7.83
Ca17883	Pectinesterase/pectinesterase inhibitor	40.57	−18.81
Ca15441	G2/mitotic specific cyclin	38.22	−9.01
Ca07832	Polygalacturonase	36.52	−6.23
Ca05687	DNA damage repair/toleration protein *DRT*	29.45	−4.69
Ca27317	*Rho-GTPase*	28.98	−63.27
Ca15305	Salicylate carboxymethyltransferase	27.82	−74.14
Ca04516	Non-specific lipid transfer protein	23.02	−3.40
Ca11089	Kinesin	22.67	−5.22
Ca05419	Leucine rich repeat receptor protein kinase	21.57	−6.81
Ca05132	Peroxidase	20.54	−11.21
Ca15364	Chaperone protein *dnaJ*	20.45	−5.32
Ca31146	Protein trichome birefringence	19.11	−15.57
Ca28965	Receptor protein kinase	17.67	−9.51
Ca08233	Basic leucine zipper	17.56	−25.54
Ca22188	Terpene synthase	16.17	−12.85
Ca13532	Arabinogalactan peptide	15.97	−123.97
Ca05547	Phospholipase D	14.44	−4.64
Ca11315	Endoglucanase	13.39	−5.21
Ca18322	Auxin efflux carrier	12.78	−7.22
Ca27258	*ABC* transporter	11.53	−14.25
Ca06284	Glucanase	11.18	−2.83
Ca05458	Auxin induced protein *IAA6*	11.02	−9.41
Ca12822	*Pectate lyase*	10.46	−6.11
Ca04109	*Condensin*	10.25	−8.20
Ca17712	*ASPARTIC PROTEASE IN GUARD CELL*	9.93	−6.34
Ca26863	Gibberellin regulated family protein	9.74	−8.39
Ca29191	Alpha-glucosidase	9.70	−9.21
Ca19842	Histone H4	9.67	−6.77
Ca21266	Glucan endo 1,3-betaglucosidase	8.64	−5.74
Ca18622	Sugar porter (*SP*) family *MFS* transporter	7.38	−5.64
Ca20846	Zinc finger	7.12	−4.21
Ca18708	Proline rich protein	7.03	−7.02
Ca13369	Allene oxide synthase	6.64	−9.66
Ca12046	alpha-L-fucosidase 2	6.41	−18.78
Ca05618	*Cdc2MsD* protein	6.36	−4.50
Ca31064	()isopiperitenol/()carveol dehydrogenase	6.08	−6.91
Ca29950	Protein *SRG1*	6.07	−11.03
Ca25807	*GDSL* lipase/acylhydrolase	5.98	−11.36
Ca30964	Lamin protein	5.66	−12.08
Ca27357	Cellulose synthase	5.51	−3.42
Ca12582	Glucomannan 4- beta-mannosyltransferase	5.41	−28.43
Ca14197	*SIEVE ELEMENT OCCLUSION B*	5.40	−4.59
Ca26452	Alkaline alphagalactosidase	5.16	−2.55
Ca14732	Betagalactosidase	4.56	−13.29
Ca13624	Histone lysine N-methyltransferase	4.32	−9.36
Ca12414	Cytochrome P450	4.22	−3.65
Ca28514	Protease inhibitor/seed storage/*ltp*	4.12	−2.78
Ca26510	*ARM REPEAT PROTEIN INTERACTING WITH ABF2*	4.09	−7.24
Ca06506	Cellulose synthase A	3.51	−9.82

#### Transcription Factors (TFs)

**Figure 11:**
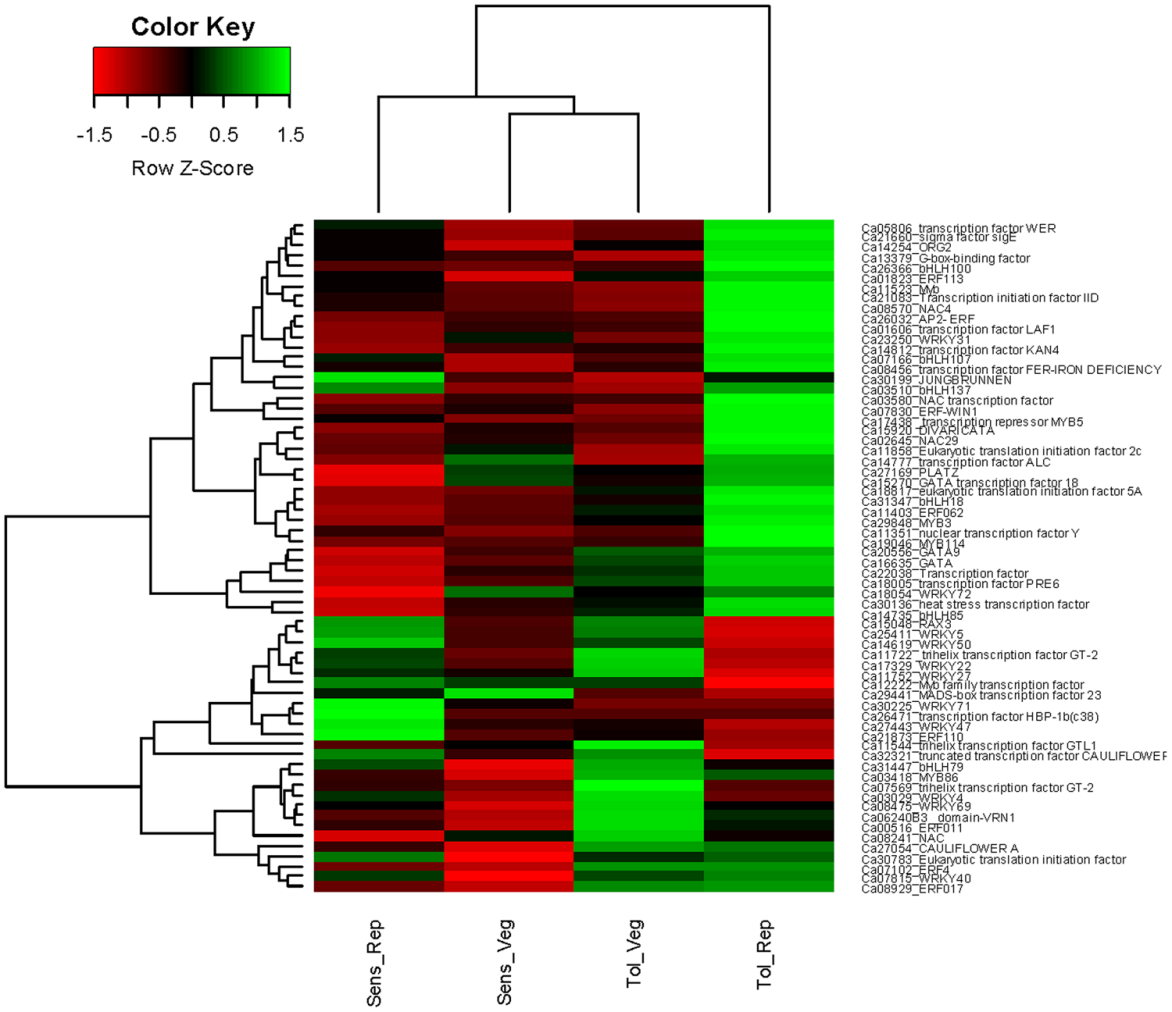
Heatmap showing differential expression of different transcription factor families amongst the tolerant and sensitive genotypes at developmental stages in response to salt stress. Both row and columns are clustered based on the gene expression values.

Transcription factors are special proteins that control the transcription of genes, and a lot of them are therefore expressed in a genotype, tissue, and stress-specific manner. A total of 151 TFs were differentially expressed, with 91 up-regulated and 60 down-regulated in response to salt stress. The most expressed TF families were *NAC*, *ERF*, *WRKY*, *bHLH*, *MYB*, trihelix-factor, *HSF* and *GATA*. It is important to note that the *NAC*, *ERF*, *WRKY*, and *HSF* were highly induced while trihelix factor was repressed in the tolerant genotype suggesting their role in stress response and plant growth processes (Fig. 11).

AP2/ERF transcription factors: A suite of ERFs has been reported to be induced in response to high salinity, osmotic stress, and drought^31^. Interestingly, ethylene-responsive transcription factor (*ERF-WIN1*) was highly induced in the tolerant genotype (Ca07830; FC: 36.25 ↑) while being repressed in the sensitive genotype (Ca07830; FC: −1.03 ↓). Another important ethylene factor is *AP2/ERF* which plays a major role in plant development *via* cell proliferation and hormonal responses during the stress^32^. An *AP2/ERF* TF was 10-fold more induced in the tolerant genotype (Ca26032; FC: 11.71 ↑) as compared to the sensitive genotype (Ca26032; FC: 1.10 ↑). The *AP2/ERF* factors are thought to bind dehydration responsive elements at the promoter of target genes and instigate salt stress response^33^ (Sakuma *et al*.^33^). Interestingly, dehydration responsive element (*DRE/CRT*) was significantly induced in the tolerant genotype (Ca13645; FC: 15.56 ↑) while being repressed in the sensitive genotypes (Ca19176; FC: −6.86 ↓).

NAC transcription factors: In addition to ethylene factors, *NAC*s are important TFs which have been reported to impart salt tolerance in *Arabidopsis thaliana*^34^, *Oryza sativa*^35^, and *Glycine max*^36^. The *NAC* TFs are involved in flower development, cell division, shoot apical meristem formation and secondary wall synthesis^37^. Many *NAC* factor transcripts were significantly induced in the tolerant genotype (Ca03580; FC: 77.70 ↑) while being repressed in the sensitive genotype (Ca15352; FC: −28.64 ↓). Significant differential expression of *NAC* factors amongst the two genotypes suggests they may contribute to salt tolerance/sensitivity in chickpea.

MYB transcription factors: *MYB*s are an important class of TFs which are widely distributed in plants and play a role in cell development, cell cycle and primary/secondary metabolism^38^. The *MYB* TFs can regulate both, positive and negative gene expression by silencing the transcription^39^. This is accomplished by the expression of *MYB*-repressor genes that control the synthesis of lignin and modulation of secondary cell wall formation in plants during the stress^40^. The *MYB* TFs were induced in the tolerant genotype (Ca24358; FC: 19.56 ↑) while being repressed in the sensitive genotype (Ca24358; FC: −6.96 ↓). Similarly, *MYB*-repressor (*MYBH*) was highly induced in the tolerant genotype (Ca17438; FC: 65.34 ↑) and repressed in the sensitive genotype (Ca27438; FC: −6.65 ↓). The effect of both these genes is essential to maintain auxin homoeostasis and their up-regulation in the tolerant genotype suggests major role in cell wall modification in order to protect the cell from osmotic pressure exerted by the salt stress^41^.

WRKY transcription factors: Another most important family of TFs extensively distributed in plant genome is *WRKY* transcription factors. They comprise of several diverse *WRKY* members which regulate the gene response to abiotic stress, root development, leaf senescence, seed germination and phytohormone signalling^25,42,43^. Different *WRKY* factors are highly tissue specific and reported to be critical for salt stress tolerance^44,45^. The *WRKY72* factor (Ca18054; FC: 26.90 ↑) was highly induced while *WRKY73* was highly repressed in the tolerant genotype (Ca21854; FC: −177.29 ↓). On the contrary, *WRKY50* factor was induced in the sensitive genotype (Ca14619; FC: 5.09 ↑) while repressed in the tolerant genotype (Ca14619; FC: −1.67 ↓). Importantly, the stress responsive genes regulated by *WRKY72* are known to be non-reponsive to salicylic acid while *WRKY50* regulates the genes which are responsive to jasmonic acid^46,47^. This suggests that various *WRKY* genes have specific role in transcriptional re-programming^48^ and the two genotypes employ different mechanisms to combat the salt stress.

Heat stress transcription factors (HSF): Other important differentially regulated TFs in response to salt stress are heat stress transcription factor (*HSF*). They have been suggested to regulate signalling pathways during salinity, oxidative and osmotic stress in seedlings^49^. Over-expression of *HSFs* in *Arabidopsis* was demonstrated to improve salt tolerance^50^. An *HSF* gene was highly induced in the tolerant genotype (Ca30136; FC: 124.49 ↑) but significantly repressed in the sensitive genotype (Ca30136; FC: −13.8 ↓). The significant differential expression of this particular *HSF* needs to be validated further to investigate its role during salt stress in chickpea.

Truncated transcription factor CAULIFLOWER D: The truncated transcription factors are known to prevent early flora and meristem development. A truncated *CAULIFLOWER D* TF was significantly down-regulated in the tolerant genotype (Ca32321; FC: −206.50 ↓) while being up-regulated in the sensitive genotype (Ca32321; FC: 20.82 ↑). Several physiological studies in chickpea have indicated that salinity delays flowering time (Turner *et al*.^8^; Khan *et al*.^5^). The down-regulation of this gene in the tolerant genotype suggests that delaying flowering time could be an important mechanism to avoid the effect of salt stress during the reproductive phase to restore pod filling process.

#### DEGs Involved In Cell Wall Modification

**Figure 12:**
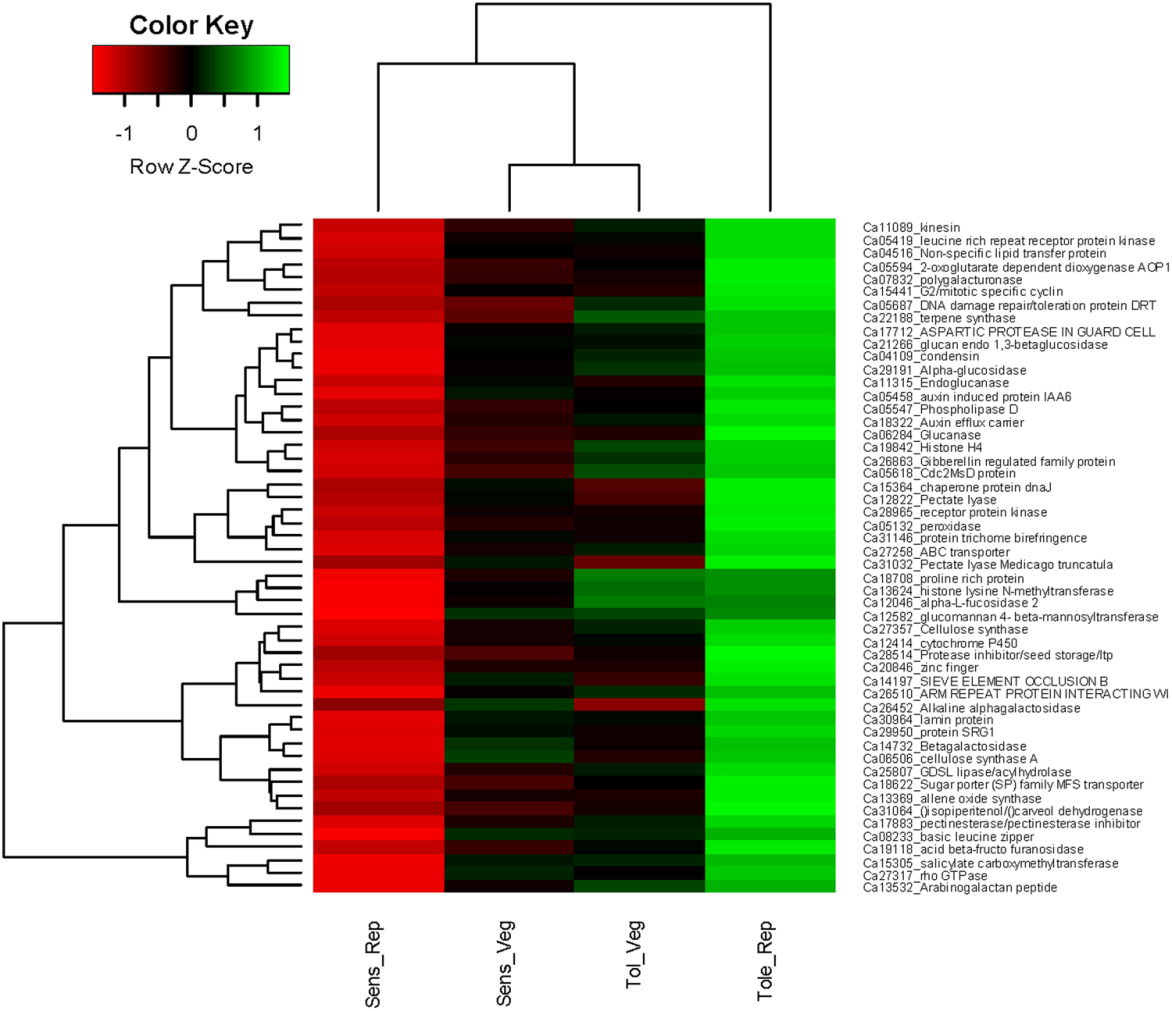
Heatmap showing differential expression of important genes amongst the tolerant and sensitive genotypes at developmental stages in response to salt stress. Both row and columns are clustered based on the gene expression values.

The plant cell wall is the first site to perceive and respond to abiotic stress^51^. The stress signal triggers cell wall remodelling so as to maintain the flexibility and protect the cell against ionic imbalance^52^. Major genes involved in cell wall organisation are dirigent protein, phosphatidylinositol phosphate kinase and lipid transfer proteins. The dirigent proteins (*DIR*s) are glycoproteins with an important role in lignan and lignin biosynthesis and are involved in secondary metabolism^53^. Interestingly, dirigent protein (Ca12786; FC: 1024 ↑), lipid-transfer protein (Ca19748; FC: 142.02 ↑), phosphatidylinositol phosphate kinase (Ca17709; FC: 77.7 ↑) and pectate lyase (Ca31032; FC: 60.96 ↑) were highly induced in the tolerant genotype but repressed at the same time in the sensitive genotype (Table 2). Among these genes, lipid transfer proteins are most important for stabilisation of membranes, cell wall organisation and signal transduction in response to biotic and abiotic stress. The lipid-transfer protein was highly induced in the tolerant genotype (Ca19748; FC: 142.02 ↑) but repressed in the sensitive genotype (Ca19748; FC: −1.42 ↓) indicating that the sensitive genotype lacks cell membrane stabilization during the adverse effect of ionic stress (Fig. 12**)**.

In a related observation, cell surface glycoproteins such as arabinogalactan peptide protein (*AGP*s) were differentially expressed in response to salt stress. These genes are wall-associated proteins and help in cell elongation, cell development and signalling^54^. Two such genes namely, arabinogalactan peptide protein (Ca13532; FC: 25.10 ↑) and proline-rich protein (Ca17660; FC: 34.77 ↑) which are involved in cell wall re-shaping were highly induced in the tolerant genotype but significantly repressed in the sensitive genotype (Ca13532; FC: −123.6 ↓).

Plant cell wall is composed of different biomolecules including cellulose, pectins and lignin. The cell wall modification under abiotic stress is accomplished by modulation in cross-linking between these polymers. Genes such as expansin and xyloglucan are involved in modulating these cross-links and regulate cell wall metabolism process. Importantly, expansin gene was highly induced in the tolerant genotype (Ca19156, FC: 88.03 ↑) but repressed in the sensitive genotype (Ca19156, FC: −2.44 ↓). Expansin and xyloglucan modifying enzymes have been reported to impart salt and drought tolerance when over-expressed in *Capsicum* and wheat in response to osmotic stress and salt stress, respectively^55^. Another important gene which is involved in cell wall metabolism is xyloglucan galactosyltransferase^56^. The xyloglucan galactosyltransferase was significantly up-regulated in the tolerant genotype (Ca00249, FC: 68.59 ↑) but down-regulated in the sensitive genotype (Ca00249, FC: −25.99 ↓). According to^57^ knock-out of xylogucan *XHT*s gene imparted more aluminium tolerance and reduced root elongation in *Arabidopsis* and they are regarded as important stress marker gene in salt, cold and drought stress^58,59^. Xyloglucans are linked to at least two cellulose molecules and are reported to remodel stomata cell walls to prevent excess water loss during the osmotic stress^56^. Cell wall remodelling promotes the plant growth and the up-regulation of expansin and xyloglucan genes in the tolerant genotype clearly suggests their activity in ‘cell wall loosening’ may impart salt stress tolerance in chickpea.

#### DEGs Involved In Hormonal Crosstalk in Response to Salt

Plants can continuously change their growth and development patterns by the action of plant hormones^60^. Hormones build up a signalling network and regulate several metabolic and transcriptional processes thus altering the physiological parameters in response to stress^61^. Differential regulation of classic hormones like auxin and ethylene suggest their major role in combating the stress. Many genes related to classic hormones like ethylene, auxin, gibberellin, salicylate and jasmonate were significantly up-regulated during the salt stress.

Ethylene-related genes: Auxin and ethylene hormones work as antagonists to regulate lateral root growth whereas they synergistically regulate root hair development and root cell elongation^62^. Recent studies have focussed on ethylene involvement in modulation of cell membrane receptors and transcription factors in salt response^63^. One such important ethylene precursor aminocyclopropane carboxylate (*ACC*) oxidase suppresses salt sensitivity in *Arabidopsis*^64,65^. Ethylene receptors like aminocyclopropane-1-carboxylate synthase were up-regulated in the tolerant genotype (Ca17276; FC: 29.65 ↑) while being highly down-regulated in the sensitive genotype (Ca17276; FC: −20.96 ↓). This indicates that ethylene-mediated downstream signalling is critically involved in stress response and is an important hormone playing a major role in salt tolerance.

ABA-related genes: It has been well characterised that the salt stress induces ABA-related genes. The accumulation of abscisic acid (ABA) hormone during salt stress helps to close the stomata and help plant acclimatise under low water availability by accumulating proteins which act as osmoprotectants for osmotic adjustment^66,67^. Interestingly, genes such as zeaxanthin (Ca20588; FC: 6.72 ↑) and *ABF2* (Ca26510; FC: 4.22 ↑) were induced in the tolerant genotypes as shown while repressed in the sensitive genotype (Ca20588; FC: −1.74 ↓). Concomitantly, an aspartic protease protein found in guard cells plays important role in osmotic adjustment during stress by signalling increased ABA sensitivity and preventing water loss from the cells^68^. This gene was significantly up-regulated in the tolerant genotype (Ca17712; FC: 10.41 ↑) while highly repressed in the sensitive genotype (Ca17712; FC: −6.32 ↓) suggesting its important role in osmotic stress regulation. One of the important ABA-responsive protein is late embryonic abundant-like protein which was 82-fold more induced in the tolerant genotype (Ca14458; FC: 252.47 ↑) as compared to the sensitive genotype (Ca14458; FC: 3.05 ↑). This gene is known to prevent protein aggregation thus protecting the plants against high salinity, desiccation and temperature^69,70^.

Auxin-related genes: Root remodelling and plant development is a major challenge during the salt stress^71^. Auxins have a major function in root remodelling, tissue development, cell elongation and apical dominance which aid in plant adaptation^72^. Several studies report auxin as the main hormone responsible for altering the growth developmental pattern and inducing genes for stress tolerance^73,74^. Several auxin-related genes such as auxin-responsive protein (Ca11225; FC: 15.4 ↑), *SAUR*-like auxin-responsive family protein (Ca15351; FC: 7.51 ↑) and auxin-induced protein (Ca05458; FC: 10.55 ↑) were induced in the tolerant genotype as shown while repressed in the sensitive genotype (Ca11225; FC: −5.02 ↓). Gradients of auxin are important for root cell differentiation, lateral growth pattering and cell elongation^75^. This is regulated by number of membrane localised proteins such as *PIN-FORMED* family of auxin efflux carrier (Ca18322; FC: 11.95 ↑), multidrug resistance P-glycoprotein (Ca30923; FC: 5.81 ↑), and *ABC* transporters (Ca27258; FC: 11.71 ↑) (Lewis *et al*., 2007; Wu *et al*., 2007) that were significantly induced in the tolerant genotype as shown. On the contrary, auxin efflux carrier was repressed in the sensitive genotype (Ca18322; FC: −7.21 ↓). Another important auxin gene, indole-3-acetic acid-amido synthetase (*GH3*) was up-regulated in the tolerant genotype (Ca08527; FC: 40.22 ↑) while being down-regulated in the sensitive genotype (Ca08527; FC: 1.61 ↓). *GH3* gene is thought to regulate expansin gene which loosens the cell wall and induces salicylate- and jasmonate-mediated disease resistance pathways^76^.

Jasmonic acid-related genes: Jasmonic acid (JA) is reported to impart salt tolerance in many different plants^77,78^. Jasmonates have important role in developmental and defense mechanisms, root growth inhibition, vegetative storage proteins (*VSP*s) accumulation, induced systemic resistance (*ISR*) and tolerance to ozone O3^79,80^. Up-regulation of jasmonic acid-amido synthetase (*JAR1*) gene in the tolerant genotype (Ca33318; FC: 41.64 ↑) indicates its role in JA-mediated defence response pathways while this gene was not expressed in the sensitive genotype. Plant hormones are crucial endogenous molecules which regulate several plant developmental processes and response to biotic and abiotic stresses^63^. The differential expression of different hormone-related genes suggests their critical role in shaping chickpea plant morphology that enables it to adapt to salt stress.

#### DEGs Involved In Root Morphogenesis

**Figure 13:**
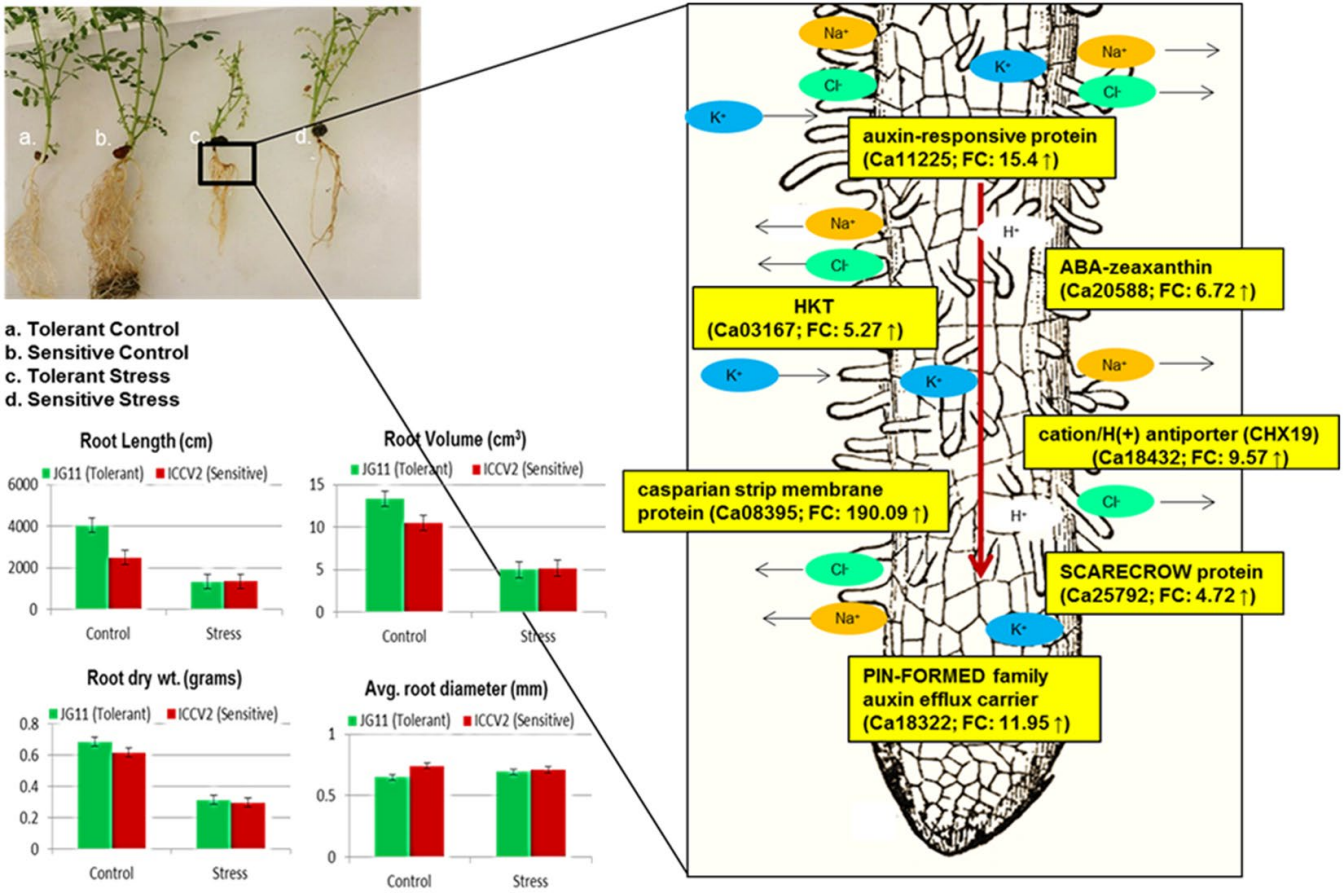
Proposed root development modulation instigated by differentially expressed genes up-regulated in tolerant genotype. Integration of phenomics and transcriptomics data showing the auxin flow controlled by *PIN-FORMED* genes regulate root agravitropism in response to salt stress.

Plants employ different sensory mechanisms to deal with high soil salinity and re-shaping the root architecture is one such important mechanism (Galvan-Ampudia and Testerink^24^). Crosstalk between classic phytohormones like auxin, ethylene, abscisic acid (ABA), salicylic acid (SA) and jasmonic acid (JA) coordinate the root growth and radial pattering^81^. Patterning in root and lateral root hair formation is important for plant adaptation during the stress^82,83^. Root endodermis is the membrane which functions in nutrient uptake, diffusion and stress resistance. Several root development genes were induced in the tolerant genotype in reponse to salt stress. One of the important root development gene such as casparian strip membrane protein was highly induced in the tolerant genotype (Ca08395; FC: 190.09 ↑) while being repressed in the sensitive genotype (Ca08395; FC: −3.68 ↓). In addition, important genes like *SCARECROW* protein (Ca25792; FC: 4.72 ↑), root meristem growth factor (Ca16049; FC: 29.85 ↑), *LONGIFOLIA* protein (Ca27082; FC: 44.94 ↑) and *RADIALIS* protein (Ca11227; FC: 4.08 ↑) which lead to formation of root endodermis were highly induced in the tolerant genotype but repressed in the sensitive genotype (Fig. 13). Further, the *COBRA* gene was highly repressed in the sensitive genotype (Ca27034; FC: −7.46 ↓). The *COBRA* gene helps in polar root expansion and its down-regulation in the sensitive genotype suggests that this important response was inhibited^84^. During salt stress, the root grows in agravitropic manner and cells expand radially^85^. These changes in lateral root development and agravitropism is mediated by auxin redistribution^86,87^. Importantly, this finding is supported by our physiological data (Figs 1 and 2), where the root volume increased in the tolerant genotype rather than root length. On the other hand, *Transparent TESTA* gene which has a role in the development of root hair in the epidermal cell layer was highly induced in the sensitive genotype (Ca31822; FC: 494.55 ↑)^88^. The bulging of root hair cells increases the influx of Ca^2+^/Na^+^ ions and up-regulation of this gene in the sensitive genotype could possibly be the reason for enhanced sensitivity to salt stress in chickpea^88,89^.

#### DEGs Involved In Network of Transmembrane Transporters

The plant’s first response to salinity is either ion-exclusion or sequestration of ions in the vacuoles leading to tissue tolerance^9,90^. Several ion-regulation pathways are integrally active in plant roots as the absorption of nutrients from soil solution requires constant inclusion/exclusion of ions. Many cation exchanger proteins were up-regulated in the tolerant genotype while down-regulated or not differentially expressed in the sensitive genotype. Cation/H^+^ antiporters are expressed in endodermis of the root and act as a channel to transport Na^+^ from endodermis to stele cells of the root eventually loading these ions in the xylem and aerial plant tissues^90,91^. One important cation/H(^+^) antiporter (*CHX19*) involved in ion-exclusion pathway was 7.3-fold more induced in the roots of the tolerant genotype (Ca18432; FC: 9.57 ↑) as compared to the sensitive genotype (Ca18432; FC: 1.37 ↑)^92^.

Plant High-Affinity Potassium Transporters (*HKT*s) are important ion channels that were recently demonstrated to selectively allow the Na^+^ and/or K^+^ transport and are therefore potential candidates for salt tolerance^93,94^. Potassium transporters are also known to unload the Na^+^ ions from xylem sap thus preventing the toxic ions from reaching and accumulating in the leaves^95^. Potassium channels also regulate import of K^+^ ions thereby balancing the K^+^/Na^+^ ratio in tissues and preventing salt stress^96^. One potassium transporter was 1.55-fold more induced in the tolerant genotype (Ca03167; FC: 5.27 ↑) as compared to the sensitive genotype (Ca03167; FC: 3.48 ↑) during the reproductive stage. This may prevent toxic Na^+^ ions from reaching the aerial parts of chickpea thereby ensuring successful seed setting and crop yield.

Another important key mechanism for ion-detoxification is vacuolar sequestration of toxic ions in the vacuole. We found a number of genes associated with vacuolar compartmentalisation of ions were differentially expressed indicating tissue tolerance through ion sequestration is possibly a main mechanism of salt tolerance in chickpea. The *NRT1-PTR* gene family (*NPF*) are identified as nitrate or di/tri-peptide transporters and involved in vacuolar compartmentalisation^97^. It was recently demonstrated to be expressed in root tissues and localised at the vacuolar membrane. An *NRT1-PTR* family gene was significantly induced in the tolerant genotype (Ca00319; FC: 95.67 ↑) while being repressed in the sensitive genotype (Ca00319; FC: −4.85 ↓). It is also known to transport phytohormones, nitrogen and secondary metabolites^97^. The ion-channels help to maintain low levels of cytosolic Na^+^ in the roots but if this mechanism fails, salinity will induce osmotic stress similar to drought^88^.

Apart from this, transmembrane water channels called aquaporins regulate the transport of fluids and are reported to play a major role during salt induced osmotic stress responses. Importantly, transmembrane major intrinsic protein (*MIP*) channels were induced in the tolerant genotype (Ca03453; FC: 54.5 ↑) while repressed in the sensitive genotype (Ca03453; FC: −2.73 ↓). They are major class of intrinsic proteins which regulate the transport of ion, water and carbohydrates. In addition to water, plants accumulate soluble osmolytes such as sugars, proline, betaine to help protect the cell against the osmotic stress^98,99^.

Other important transporters with *ATPase* activity to allow selective transport across the transmembrane were highly induced in the tolerant genotype. Examples are cyclic nucleotide-gated ion channel (Ca16646; FC: 37.53 ↑), metal transporter *Nramp* (Ca21750; FC: 5.57 ↑), extracellular ligand-gated ion channel protein (Ca13670; FC: 33.82 ↑), and white-brown complex protein (Ca26763; FC: 294.06 ↑). These were either not expressed or repressed in the sensitive genotype; example, cyclic nucleotide-gated ion channel (Ca16646; FC: −4.14 ↓). These results indicate that the two genotypes employ different approaches to deal with salt stress. Further, there exists strong evidence that ion-exclusion mechanism may confer salt tolerance in chickpea. This would be further validated and utilised to improve salt tolerance in chickpea.

#### Cellular Ion Homeostasis Through Metallozymes

Metallothioneins are an important class of intracellular metal binding enzymes involved in detoxification of heavy metals and maintaining ion-homeostasis. They often have a high affinity towards heavy metals^100,101^ and are known to provide tolerance against heavy metal ion toxicity^102^. Several metallozymes were found to be induced in the tolerant genotype suggesting their major role during the salt stress response. For instance, Type1 metallothionein (*MT1*) was induced in the tolerant genotype (Ca04758; FC: 4.69 ↑) while the expression of this gene class was not observed in the sensitive genotype. Apart from this, several metal proteins were highly induced only in the tolerant genotype and these include monothiol glutaredoxin (Ca27338; FC: 891.44 ↑), purple acid phosphatase (Ca01925; FC: 23.42 ↑), and metal tolerance protein (Ca08940; FC: 6.32 ↑). On the contrary, metal ion-binding gene (Ca27452; FC: −4.02 ↓) and heavy metal-detoxification gene (Ca29520; FC: −13.08 ↓) were highly repressed in the sensitive genotype. Another important metal enzyme is multi-copper oxidase protein which was induced in the tolerant genotype (Ca15190; FC: 4.37 ↑) while repressed in the sensitive genotype (Ca15190; FC: 3.89 ↓). Multi-copper oxidase gene is thought to oxidise copper and similar ions which could be toxic to cells. Its up-regulation in the tolerant genotype suggests a role in reducing metal toxicity during the salt stress. The functions of these metal enzymes in metal detoxification are well demonstrated in *Arabidopsis*^103^. However their role in plant metal detoxification during salt stress still remains to be established. Nevertheless, their differential expression in this study suggests that they play an important role in metal metabolism or detoxification during the salt stress^103,104^.

## Methods

### Plant Material

Two chickpea genotypes, *desi* JG 11 (salt tolerant) and *kabuli* ICCV 2 (salt sensitive), were subjected to salt stress in the glasshouse^1^. The physiological experiment was set-up in a Random Complete Block Design (RCBD) comprising three biological replicates of each genotype. The seeds were surface sterilised using 70% ethanol followed by three rinses with MilliQ water. The seeds were sprouted in Petri dishes in dark and moist conditions until radicle emergence. The etiolated seedlings were sown in 10.5 inches diameter pots filled with 9.5 kg of pasteurized sand soil mix. In the treatment pots, two adaptive doses of salt at the rate of 40 mM NaCl were added twice during the life cycle of plant; one at 10 days after sowing and another at the flowering stage. Initial salt treatment included mixing sodium chloride in the soil (1.75 g per kg of soil) before filling the pots^6,8^. Pots were sealed with sturdy tape to prevent the leakage of salt. To avoid water retention in pots, the soil field capacity was measured gravimetrically and pots were maintained at 80% field capacity throughout the experiment. To monitor the salt concentration, soil electrical conductivity was checked and maintained below ~1 dS/m considering the intrinsic salt sensitivity of chickpea. Control plants were grown normally in 9.5 kg of pasteurized sand soil mix and maintained at 80% field capacity. Plants were routinely watered and root tissues were harvested at the vegetative and reproductive stages and immediately frozen in liquid nitrogen.

### RNA Isolation and Library Preparation

Total RNA was isolated using Qiagen RNeasy kit (GmBH, Germany). The frozen root tissue was ground to fine powder and weighed to add calibrated volumes of lysis buffer. Finally, RNA was eluted in 60 µl of RNase-free water. RNA was quantified using Nanodrop (NanoDrop™ Lite Spectrophotometer, Thermo Fisher Scientific) and qualitatively analysed on Bioanalyzer 2100 (Agilent Technologies). Only RNA with RIN values > 7 were chosen for mRNA enrichment.

### mRNA Enrichment

From 1 µg of total RNA as starting material, poly(A^+^) mRNA was isolated using Dynabeads mRNA purification kit (Thermo Fisher Scientific, USA).

### RNA-Seq Library Preparation

A total of 24 RNA-Seq libraries (2 genotypes x 2 time-points x 2 conditions x 3 biological replicates) were prepared from 100 ng of poly(A^+^) mRNA as starting material using Ion total RNA seq kit v2 (Thermo Fisher Scientific, USA). The mRNA was chemically fragmented followed by cDNA synthesis and adapter ligation. The unstranded library molecules were analysed on Bioanalyzer 2100 (Agilent Technologies) for peak size selection and region molarity calculations. Four uniquely barcoded libraries were pooled to an equimolar concentration of 0.8 nM and enriched with ion sphere particles (ISPs) using streptavidin beads and Ion One-Touch (Thermo Fisher Scientific, USA). The charged ISPs were loaded on to the Ion-Chip and ~20 million single-end 100 bp reads were sequenced per library.

### RNA-Seq Data Processing And Differential Gene Expression Analysis

The raw fastq sequences were trimmed for adapters and read length using the q-trimming program. All reads less than 30 bp and poor quality bases were discarded. The reference genome was indexed using bowtie2-intel/2.1.0^105^. The structural annotation file (gff3) was indexed using tophat to generate a known transcript file. Ninety-two per cent clean reads were mapped against the chickpea (CDC Frontier) reference genome using tophat-gcc/2.0.13^106^. The gene counts were extracted from accepted hit bam files using HTSeq (Anders *et al*., 2014). The gene counts were used to identify differentially expressed genes using the EdgeR tool (Robinson *et al*., 2010), from Blast2GO PRO software. A gene was considered to be differentially expressed if log_2_ fold change was >1 and false discovery rate (FDR) was <0.05 (padj –BH method)^107^.

### Data availability

The raw fastq files and gene count files for all samples including three biological replicates are submitted to the GEO databases under the accession GSE110127.

## Conclusions

The availability of chickpea genome has provided a new opportunity to study the candidate genes involved in salt tolerance mechanisms in chickpea. The integration of phenotypic and genomic data in this study has provided a better understanding of salt tolerance mechanism in chickpea. A suite of genes important for salt tolerance were differentially expressed between the two genotypes suggesting that the tolerant genotype has evolved a more robust mechanism of salt tolerance in comparison to the sensitive genotype. Important candidate genes related to salt tolerance such as Cationic peroxidases, Expansins, Lipid transfer proteins, Cation/H + exchangers, Casparian strip membrane proteins, *PIN-FORMED* and Type1 metallothionein are related to physiological processes including cell wall modification, root growth modulation, ion-exclusion and phytohormone signalling. The pattern of gene expression suggested that salt induced osmotic stress followed by oxidative and ionic stress in the genotypes. However, the tolerant genotype utilised a number of genes to detoxify the effect of salt stress through highly induced actions of the above mentioned candidate genes. In conclusion, both the genotypes possess transcriptomic variation and deploy different molecular mechanisms through either stress-avoidance or stress-tolerance in response to salt stress. The candidate genes should be functionally validated using modern gene-knock out techniques such as CRISPR/Cas9 system before being employed in molecular breeding or genetic engineering for salt tolerance.
